# Maternal Childhood Trauma and Offspring Hypothalamic–Pituitary–Adrenal Axis Function from Infancy to 6 Years of Age

**DOI:** 10.1002/dev.70029

**Published:** 2025-02-17

**Authors:** Lisa Loheide‐Niesmann, Roseriet Beijers, Carolina de Weerth, Maaike Cima

**Affiliations:** ^1^ Behavioural Science Institute Radboud University Nijmegen the Netherlands; ^2^ Department of Cognitive Neuroscience Radboud University Medical Center, Donders Institute for Brain, Cognition and Behaviour Nijmegen the Netherlands; ^3^ VIGO, Juvenile Youth Institutions (YouthCarePLUS) Nijmegen the Netherlands

**Keywords:** maternal childhood trauma, adverse childhood events, intergenerational transmission, cortisol reactivity, circadian cortisol, HPA axis

## Abstract

Childhood trauma experiences can carry over to the next generation, affecting the health and behavior of survivors’ children. However, the mechanisms underlying these intergenerational effects of childhood trauma are not yet clear. One mechanism may be changes in children's hypothalamic–pituitary–adrenal (HPA) axis. This preregistered longitudinal study examined associations between 170 mothers’ childhood trauma experiences (maltreatment, family and peer violence) and their children's cortisol reactivity and total circadian cortisol output at 12 months and 6 years of age. Multilevel regression analyses revealed that maternal childhood trauma was not significantly associated with child cortisol reactivity or total circadian cortisol output, neither at 12 months nor at 6 years of age. Thus, we found no evidence in this community sample that maternal childhood trauma impacts young children's HPA axis functioning. Exploratory analyses revealed moderation effects of maternal prenatal psychopathology and prenatal circadian cortisol slope: in mothers with high prenatal psychopathology or circadian cortisol slope, maternal childhood trauma was positively associated with child total circadian cortisol output, while this association was negative in mothers with low psychopathology or circadian cortisol slope. Future research should replicate these findings in older children and more severely trauma‐exposed populations and further explore moderators of this intergenerational association.

## 1 Introduction

Traumatic childhood experiences can have severe, long‐lasting mental health consequences for survivors (Lewis et al. 2019), but also for survivors’ children. Children of childhood trauma‐exposed mothers exhibit more mental health problems, including internalizing and externalizing disorders (Loheide‐Niesmann, Riem, and Cima 2024; Su, Arcy, and Meng 2022). However, little is known about the mechanisms behind this intergenerational transmission of the effects of childhood trauma. One mechanism hypothesized to underlie the link between maternal childhood trauma and child behavior is change in child stress system functioning, including the hypothalamic–pituitary–adrenal (HPA) axis. This study examines whether maternal childhood trauma is associated with young children's cortisol reactivity and total circadian cortisol output, as biomarkers of HPA axis functioning.

The HPA axis, with its end‐product cortisol, is an important regulator of the physiological stress response (de Weerth et al. 2013). Additionally, the HPA axis displays a basal diurnal function, releasing glucocorticoids throughout the day. This circadian rhythm is characterized by high morning cortisol concentrations, which decline throughout the day (Hostinar and Gunnar 2013). Dysregulation of both cortisol reactivity to stressors and basal diurnal cortisol have been related to mental health problems such as internalizing and externalizing behavior. Lower basal cortisol concentrations have been related to more externalizing problems (Alink et al. 2008) and a flatter diurnal slope has been related to both more internalizing and more externalizing problems (Adam et al. 2017; Saridjan et al. 2014). Findings on cortisol reactivity are mixed; externalizing behavior has been associated with both lower and higher cortisol reactivity or was found to not be associated with cortisol reactivity at all (Alink et al. 2008; Figueiredo et al. 2020). Similarly, internalizing behavior has been associated with both increased (Hartman et al. 2013) and blunted cortisol reactivity (Sun et al. 2022). Overall, behavioral problems such as internalizing and externalizing behavior have thus frequently been associated with HPA axis dysregulation, which underlines the plausibility of the HPA axis as a potential mechanism through which the effects of mothers’ experiences of childhood trauma might affect the behavior and mental health of their children.

The fetal HPA axis starts to develop in utero, undergoing remarkable growth and organization (Howland, Sandman, and Glynn 2017). This development continues during the early postpartum years, as evidenced by variation in cortisol stress reactivity and circadian cortisol output throughout development (Howland, Sandman, and Glynn 2017). Moreover, the normative cortisol circadian rhythm is not yet present after birth, but emerges during the first year of life and continues developing throughout toddlerhood (de Weerth, Zijl, and Buitelaar 2003; Simons et al. 2015). During its pre‐ and postnatal development, the HPA axis is susceptible to environmental influences (Howland, Sandman, and Glynn 2017). Consequently, it could be during these sensitive, developmental periods that maternal childhood trauma and its sequelae could affect children's HPA axis development and functioning.

This transmission of the effects of maternal childhood trauma onto child HPA axis functioning could occur through multiple pathways before conception, during pregnancy, and/or postnatally. Before conception, childhood trauma could potentially result in epigenetic changes in the mother's germ cells, which are vulnerable to environmental influences until puberty (Yehuda and Lehrner 2018). Such epigenetic changes could then affect fetal HPA axis development and thereby postnatal HPA axis functioning in children of mothers with childhood trauma experiences. To date, however, epigenetic inheritance through the maternal germ line has not yet been empirically supported (Lindsay et al. 2020).

During pregnancy, alterations in mothers’ own HPA axis functioning could affect fetal HPA axis development (Buss et al. 2017). Childhood trauma can result in HPA axis dysregulation in survivors (De Bellis and Zisk 2014) and many common sequelae of childhood trauma—such as psychiatric disorders, including major depression and posttraumatic stress disorder (Mandelli, Petrelli, and Serretti 2015; Pratchett and Yehuda 2011), or increased smoking behavior or substance use (M. E. Roberts et al. 2008; S. Zhang et al. 2020)—have been associated with HPA axis dysregulation as well (Cowen 2010; De Bellis and Zisk 2014; Majewska 2002; Rohleder and Kirschbaum 2006; Schumacher et al. 2019). These changes in HPA axis functioning in nonpregnant survivors are likely present in pregnant women as well, as evidenced by previous findings of an increased cortisol awakening responses in pregnant women with more severe trauma and adversity exposure (Bublitz and Stroud 2012). Such increased maternal cortisol concentrations can, in turn, partially pass through the placenta and affect fetal cortisol concentrations and fetal development of the HPA axis and brain regions involved in HPA axis regulation (Beijers, Buitelaar, and de Weerth 2014; Entringer, Buss, and Wadhwa 2015). These alterations in fetal HPA axis development may subsequently result in alterations in postnatal HPA axis functioning in children of childhood trauma‐exposed mothers.

Moreover, maternal HPA axis dysregulation and increased maternal stress, for example, resulting from sequelae of maternal childhood trauma, such as psychiatric disorders, might result in an unfavourable intrauterine environment for the fetus, which could lead to the de novo production of epigenetic marks (Buss et al. 2017; Lindsay et al. 2020). Indeed, previous research has found associations between maternal stress during pregnancy, in the form of trauma exposure or a psychiatric disorder, to be associated with epigenetic alterations of the glucocorticoid receptor gene (NR3C1) (Palma‐Gudiel et al. 2015; Yehuda et al. 2014). Such alterations could then affect HPA axis development and thus postnatal HPA axis functioning in children of childhood trauma‐exposed mothers.

Postnatally, mothers continue sending physiological signals to their child through the biological constituents within their milk, which might impact children's neurobiological and behavioral development, including their HPA axis development (de Weerth et al. 2022). In line with this, breast milk of mothers experiencing more stress is found to contain higher cortisol concentrations (Browne et al. 2019; Vacaru et al. 2023). Finally, maternal childhood trauma may influence child HPA axis functioning postnatally by affecting the environment children live in, as childhood trauma‐exposed mothers are more likely to utilize harsh and insensitive caregiving practices (Bert, Guner, and Lanzi 2009). Children of childhood trauma survivors might thus experience more stressful caregiving environments, to which children's HPA axis may adapt as they mature (Yehuda and Lehrner 2018).

While maternal childhood trauma might thus affect child HPA axis functioning through multiple mechanisms, empirical evidence about this intergenerational association is just beginning to emerge. Results of studies on the association between maternal childhood trauma and child cortisol stress reactivity have been mixed. Three studies found significantly flatter cortisol stress reactivity curves in children of trauma‐exposed mothers, with children ranging in age from 1 month to 5 years (Khoury et al. 2021; Parade et al. 2019; Robinson 2016). Notably, Parade et al. (2019) only found a significant effect in 1‐month‐olds but not in 1‐day‐olds, and Robinson (2016) only found a significant effect for childhood sexual abuse but not for physical abuse or total childhood trauma scores. However, three other studies examining children between the ages of 4 and 44 months found no significant association between maternal childhood trauma and child cortisol reactivity (Brand et al. 2010; Lindrose 2021; Schlueter 2017). Finally, two studies found indirect or interaction effects only: Thomas et al. (2018) reported an indirect effect of more maternal adverse childhood events (ACEs) on higher infant cortisol reactivity, mediated by maternal HPA axis functioning during pregnancy and moderated by maternal social support. And Hertz (2019) found that maternal child maltreatment was associated with better child cortisol recovery, that is, a quicker return to baseline concentrations following stress exposure, but only in children of mothers with high trait mindfulness levels.

To the best of our knowledge, only three studies have examined the association between maternal childhood trauma and child circadian cortisol output. These studies seem to suggest a connection between mothers’ childhood trauma experiences and their children's circadian cortisol output: Hendrix et al. (2022) found more severe maternal childhood maltreatment experiences to be related to greater cortisol output in 3‐month‐olds, even after controlling for mothers’ prenatal distress. Similarly, Schlueter (2017) found higher noon and bedtime cortisol concentrations in 5–44‐month‐olds of mothers with 6 or more ACEs, compared with children of mothers with fewer than 6 ACEs. When using the number of mothers’ ACEs as a continuous predictor, however, the association between ACEs and child cortisol concentrations was nonsignificant. Additionally, Hillmann et al. (2021) reported elevated morning cortisol in 6–11‐year‐olds of childhood maltreatment‐exposed mothers with a lifetime mental disorder compared with children of childhood maltreatment‐exposed mothers with no lifetime mental disorder.

In sum, research on the association between maternal childhood trauma and child cortisol concentrations focused mostly on cortisol reactivity and provided mixed findings, whereas studies examining child circadian cortisol are scarce. Further research is thus needed to clarify the mixed findings regarding child cortisol reactivity and to add to the current, scarce knowledge on the potential link between maternal childhood trauma and child circadian cortisol. Moreover, previous studies mainly investigated infants or toddlers and largely neglected school‐aged children. Given that previous studies examining links between adversity and HPA axis function in children have found the nature of these associations to change across child age (e.g., White et al. 2017), it appears crucial to examine the link between maternal childhood trauma and child HPA axis function across ages, in order to gain a more comprehensive understanding of the nature of these potential associations.

The present study therefore investigated associations between maternal childhood trauma, defined as experiences of maltreatment (i.e., abuse or neglect) or family and peer violence before the age of 19 years, and child cortisol stress reactivity and total circadian cortisol output. We measured cortisol reactivity and diurnal cortisol when children were 12 months old and again at 6 years of age. In line with our preregistration (https://osf.io/t9vd8), we expected maternal childhood trauma to be associated with child cortisol stress reactivity and diurnal cortisol, both at 12 months and 6 years of age. Given the scarcity of previous research, we formed no specific hypotheses about the direction of these associations, that is, about whether maternal childhood trauma was associated with higher or lower cortisol concentrations.

In addition to our preregistered analyses, we also explored the associations between different subtypes of childhood trauma and child HPA axis functioning, as previous studies found associations between maternal childhood trauma and child HPA axis function only for specific types of childhood trauma (Robinson 2016). Moreover, we explored whether the association between maternal childhood trauma and child HPA axis function could be moderated by maternal prenatal psychopathology, prenatal HPA axis functioning, and breastfeeding duration. These factors may interact with childhood trauma conferring additional risk for the child (i.e., maternal prenatal psychopathology and alterations of prenatal HPA axis functioning) or protecting the child from potential effects of maternal childhood trauma (i.e. longer breastfeeding durations). Maternal psychopathology has been previously found to moderate associations between maternal childhood trauma and school‐aged children's HPA axis functioning (Hillmann et al. 2021). Moreover, both pregnancy HPA axis functioning and later breastfeeding have been theorized to be involved in the potential pathways from maternal childhood trauma to child HPA axis function (Yehuda and Lehrner 2018). Additionally, maternal HPA axis functioning during pregnancy has previously been found to moderate associations between mothers’ experiences of childhood trauma and their children's behavioral problems (Thomas‐Argyriou et al. 2020).

## 2 Methods

### 2.1 Participants

This study was part of an ongoing longitudinal project on children's psychobiological development (BIBO study; Basal Influences on Child Development), which received ethical approval from the Ethics Committee Social Sciences of the Radboud University (ECG/AvdK/07.563). A total of 193 women were recruited through midwife practices in and around Nijmegen (the Netherlands) and provided written consent for participating in the BIBO study (for further details, see Beijers et al. 2011). Inclusion criteria included a healthy, singleton pregnancy, no self‐reported drug use or physical or mental health problems, and Dutch language fluency. Of those women, 170 mothers completed questionnaires on their childhood trauma experiences; these mothers and their children (44.7% girls) were therefore included in the current study. Mothers were on average 32.54 years old (SD = 3.68) at delivery, were predominantly born in the Netherlands (94.1%), married or cohabitating (98.8% during pregnancy, 95.5% when children were 6 years old), and highly educated (78.7% attended college/university).

### 2.2 Procedure

This study used data collected during pregnancy and the first years of children's lives, when children were 12 months, 6 years, and 8 years old. During late pregnancy, mothers completed questionnaires about their current mental health and provided saliva samples for circadian cortisol analyses, taken on 2 consecutive days at predefined time points. Breastfeeding duration was assessed weekly during the first 6 postnatal months and again at 12 months.

At 12 months of age, children and their mothers were invited to the laboratory of the Radboud University, where the Strange Situation Procedure (SSP; Ainsworth et al. 1978) was carried out and saliva samples were taken. The SSP is considered a mild psychological stressor for infants, since it includes the introduction to a new environment (the university laboratory), a stranger, and two 3‐min separations from the mother (Jansen et al. 2010). At home, prior to the laboratory visit, mothers collected child saliva samples on 2 days, at predefined time points.

Around the time of the children's 6th birthday, mothers collected saliva samples of their children on 2 days as well. Additionally, a school visit took place during which children were tested in a mobile laboratory near the child's school (or at home, *N* = 6). School visits took place on a regular school day and started between 12:15 and 15:15 in the afternoon. Children were asked to refrain from eating, drinking, and physical activity in the 30 min before the school visit. If children were ill on the planned testing day, their school visit was rescheduled.

During the school visit, children were exposed to the Children's Reactions to Evaluation Stress Test (CREST; de Weerth et al. 2013), which consists of three forced‐failure tasks containing elements of uncontrollability and unpredictability and is performed in front of a judge (for further details, see de Weerth et al. 2013; Simons, Cillessen, and de Weerth 2017). The entire procedure takes 20 min and was followed by a 25‐min recovery period. Finally, children took part in another 25 min of tasks not relevant to this current study. Saliva samples were taken throughout the entire procedure. Additionally, when children were 8 years old, mothers filled out questionnaires, including questionnaires about their own adverse childhood experiences.

### 2.3 Measures

#### 2.3.1 Cortisol Stress Reactivity

At 12 months, saliva samples were taken during the laboratory visit. Samples were taken upon arrival at the laboratory (C1) and 25 (C2), 40 (C3), and 60 min (C4) after the second mother–child separation during the SSP. While C1 represents baseline, prestressor cortisol concentrations, C2 t C3 reflect reactivity cortisol concentrations, and C4 reflects recovery.

At 6 years, saliva samples were taken during the school visit. Samples were taken from all children before the start of the CREST (C1) and 15 (C2), 25 (C3), 35 (C4), 50 (C5), and 58 min (C6) after the start of the CREST. C1 and C2 represent baseline cortisol concentrations, C3 and C4 were obtained to measure cortisol concentrations in response to the stressor, and C5 and C6 represent recovery cortisol concentrations.

#### 2.3.2 Cortisol Circadian Rhythm

When children were 12 months old, mothers collected eight saliva samples of their children during 2, preferably consecutive, days on which their child did not attend childcare. When children were 6 years old, mothers collected eight saliva samples of their children, four per day, during 2 weekend days.

Saliva samples were taken at the following predefined time points: immediately after the child had woken up (T1), at 11:00 (T2), at 15:00 (T3), and at 19:00 (T4). For both the 12 month and the 6‐year assessment, cortisol concentrations from both assessment days were averaged to combined cortisol concentrations, separately for the T1, T2, T3, and T4 measures.

#### 2.3.3 Cortisol Analyses

All saliva samples were collected using eye sponges (BD Visispeare, Waltham, MA; de Weerth et al. 2007). Eye sponges were centrifuged to obtain the saliva, which was then stored in a freezer (−25°C). Subsequent cortisol analyses were carried out at the Laboratory of Endocrinology of the University Medical Center Utrecht, using an in‐house competitive radioimmunoassay with a polyclonal anticortisol–antibody (K7348) and [1,2‐ 3H(N)]‐hydrocortisone (PerkinElmer NET396250UC) as a tracer. The lower limit of detection was 1.0 nmol/L and interassay and intra‐assay variations were <10%.

#### 2.3.4 Maternal Childhood Maltreatment

Mothers’ childhood maltreatment experiences were retrospectively assessed when their children were 8 years old, by using the 25‐item Childhood Trauma Questionnaire (CTQ; Bernstein and Fink 1988). The CTQ assesses five types of childhood maltreatment, namely physical, sexual, and emotional abuse as well as physical and emotional neglect. For the analyses, a total maltreatment sum score and sum scores for each of the five subscales of maltreatment were calculated. In previous studies, the CTQ has shown very good reliability (*α* = 0.97; Bernstein et al. 1997) and in the present study, reliability was good (*α* = 0.88).

#### 2.3.5 Maternal Childhood family and Peer Violence Experiences

Mothers’ adverse childhood family and peer violence experiences were assessed when their children were 8 years old, by using parts of the Adverse Childhood Experiences International Questionnaire (ACE‐IQ; World Health Organization (WHO) 2018). Specifically, we used the family dysfunction items of the ACE‐IQ *Family violence* subscale, while excluding the subscale's questions referring to experiencing child abuse, since these experiences were covered by the CTQ. Furthermore, we used two of the three *Peer violence* items (namely “how often were you bullied?” and “how often were you in a physical fight”). The *Community violence* and *War/collective violence* subscales from the ACE‐IQ were not used, since they were deemed not applicable to our study population. The ACE‐IQ has shown satisfactory validity (Kidman et al., 2019) and good test–retest reliability (ICC = 0.90; Ho et al. 2019). For the analyses, the number of adverse childhood (family and peer violence) experiences (ACEs) were calculated for each participant.

#### 2.3.6 Prenatal Psychopathology

Around the 37th week of pregnancy (*M* = 37 weeks, 1.9 days; SD = 7.1 days), mothers completed the state subscale of the State‐Trait Anxiety Inventory (STAI; Spielberger 1983; van der Ploeg, Defares, and Spielberger 1981), and the Edinburgh Postnatal Depression Scale (EPDS; Cox, Holden, and Sagovsky 1987). The 20‐item STAI assesses feelings of general anxiety, while the 10‐item EPDS assesses symptoms of depression. The STAI and the EPDS have shown good internal consistency in previous research (STAI: *α* = 0.91; Barnes, Harp, and Jung, 2002; EDPS: *α* = 0.84; Matijasevich et al. 2014) and in the current study (STAI: *α* = 0.90; EPDS: *α* = 0.99). Sum scores were calculated separately for the STAI and the EPDS. These sum scores were standardized and then averaged to create a composite prenatal psychopathology variable, with higher values indicating more symptoms of psychopathology.

#### 2.3.7 Prenatal Circadian Cortisol

During pregnancy (*M* = 37 weeks, 0.8 days; SD = 9.4 days), mothers collected diurnal saliva samples on 2 consecutive days. Saliva samples were taken at the following predefined time points: immediately after waking up (T1), 30 min after awakening (T2), at 12:00 (T3), at 16:00 (T4), and at 21:00 (T5) (for further details regarding cortisol measurement and analyses, please see Beijers et al. 2010). Cortisol concentrations from both assessment days were averaged to create combined cortisol concentrations, separately for the T1, T2, T3, T4, and T5 measures. In line with previous research using the BIBO study sample (Beijers et al. 2010; Tollenaar et al. 2011), we operationalized mothers’ physiological distress by calculating evening cortisol (T5), with higher evening cortisol indicating less recovery from daily stressors (Dahlgren et al. 2008), and circadian cortisol slope (T1 minus T5), with smaller slopes indicating potential cortisol dysregulation (Wolf, Nicholls, and Chen 2008).

#### 2.3.8 Breastfeeding Duration

Breastfeeding duration was assessed weekly during the first 6 postnatal months and again at 12 months. For the current study, breastfeeding duration was operationalized as the number of months during which mothers had breastfed their children during the first year.

#### 2.3.9 Confounders

Analyses were controlled for child sex (assigned at birth), since sex differences in the HPA axis have been consistently reported in the literature (Panagiotakopoulos and Neigh 2014). Additionally, we controlled for maternal level of education (eight levels, ranging from primary school to university education), since maternal education has been previously related to maternal HPA axis functioning during pregnancy (Chen et al. 2010), glucocorticoid concentrations of human breastmilk (Pundir et al. 2019), children's general health and development (Davey et al. 2015; Knickmeyer et al. 2017), and infants’ cortisol concentrations (Luecken et al. 2013).

### 2.4 Analyses

#### 2.4.1 Missing Data

Of the 170 participating mother–child dyads, 167 mothers completed the CTQ and all mothers completed the ACE‐IQ. A total of 166 children participated in a stress test at 12 months to assess cortisol reactivity and 145 children participated in the stress test at 6 years. At both time points, the data of two children had to be excluded, due to less than half of their saliva samples being analyzable. Also, 141 and 139 mothers collected circadian saliva samples from their children at 12 months and 6 years, respectively. At 12 months, one child had to be excluded due to less than half of their saliva samples being analyzable and at 6 years, two children had to be excluded due to insufficient analyzable saliva samples.

Regarding the exploratory moderator variables, six mothers did not complete the psychopathology questionnaires during pregnancy, 33 mothers did not provide (analyzable) saliva samples during pregnancy, and two mothers did not report on their breastfeeding behavior during the first postpartum year.

#### 2.4.2 Preregistered Statistical Analyses

First, descriptive statistics of and correlations between all study variables were calculated. All outliers deviating from the variable mean by more than 3 standard deviations were winsorized to a value of 3 standard deviations from the mean.

To examine whether maternal childhood trauma was associated with offspring cortisol concentrations, a series of four multilevel regression‐based analyses were conducted, separately for the cortisol circadian rhythm at 12 months (Model 1) and at 6 years (Model 2) as well as cortisol reactivity at 12 months (Model 3) and at 6 years (Model 4). For all models, the cortisol measurement time points (Level 1) were nested within children (Level 2), with cortisol concentrations as the outcome. Calculating the intraclass correlation (ICC) using a null model resulted in ICC's of 0.00 for both circadian models and in ICC's of 0.48 and 0.72 for the cortisol reactivity models (Model 1: 0.00; Model 2: 0.00; Model 3: 0.48; Model 4: 0.72), indicating that between 48 and 72% of variance in cortisol reactivity concentrations were related to differences between children. Despite the low ICC's for both circadian models, we decided to carry out multilevel analyses for both circadian variables, due to the multilevel structure present in the total circadian cortisol output data (Nezlek 2008). We also conducted additional sensitivity analyses for both total circadian cortisol output variables, however, which are described below. We built up our cortisol reactivity and circadian cortisol output models by adding variables one‐by‐one. After adding a new variable, improvements to the model were assessed with the Likelihood‐ratio test. If a variable did not significantly improve a model, it was excluded from the model. First, time was entered into the models as a random factor (thus, allowing interindividual variability in the intercept and slope of the regression line). For the cortisol reactivity models (Models 3 and 4), the squared time variable (time^2^) was also entered as a next step, since the cortisol stress response, consisting of an increase and a subsequent decrease in cortisol, should be better described by a quadratic rather than a linear model. Then, the potential confounders *child sex* and *maternal level of education* were added to the model, followed by the predictors *maternal childhood maltreatment* and *maternal childhood family and peer violence experiences* as well as the interactions between each predictor and time.

#### 2.4.3 Deviations from Preregistration

##### 2.4.3.1 Sensitivity Analyses for the Preregistered Analyses

Given the low ICC's of both total circadian cortisol output models, we also carried out multiple regression analyses. To do so, we calculated two new circadian cortisol outcome variables, separately for circadian cortisol at 12 months and 6 years: children's area under the curve to the ground (AUCg, using all four cortisol measures per day; Pruessner et al., 2003), representing the total amount of cortisol during the day, and the slope (T1 minus T4), the cortisol decline from morning to evening. Both AUCg and slope were calculated separately for the 2 days of circadian cortisol assessment and were then averaged across days (Simons et al. 2015). Using regression analyses, we then analyzed whether maternal childhood maltreatment or maternal childhood family and peer violence experiences were significantly associated with children's AUCg and slope at 12 months and 6 years. Similarly, we also conducted sensitivity analyses for the cortisol reactivity measures at 12 months and 6 years. To do so, we calculated the highest poststressor cortisol concentrations (T2 or T3 at 12 months; T3 or T4 at 6 years; Beijers, Riksen‐Walraven, and De Weerth 2013), separately for cortisol reactivity at 12 months and at 6 years, and analyzed whether any of the two maternal childhood trauma variables were significantly associated with children's highest peak at 12 months or 6 years, while controlling for cortisol concentrations at baseline (T1 at 12 months; the mean of T1 and T2 at 6 years). All regression analyses were adjusted for child sex and maternal level of education.

Given that cortisol reactivity at 6 years was assessed with two measures each for baseline, peak, and recovery cortisol concentrations, we selected only the lowest baseline, highest peak, and lowest recovery measure (as done earlier by Simons et al. 2019). We performed our multilevel analyses again with these three values only, instead of the full six measurements.

##### 2.4.3.2 Moderation Analyses

In response to reviewers’ suggestions and based on previous literature suggesting that maternal prenatal psychopathology (Hillmann et al. 2021), maternal prenatal HPA axis functioning (Thomas‐Argyriou et al. 2020), and breastfeeding duration (Yehuda and Lehrner 2018) might exert moderating effects, we also deviated from our preregistration by examining whether these variables moderated the association between maternal childhood trauma and child HPA axis activity. We exploratorily added these variables and the interactions between these variables and the maternal trauma variables to the preregistered multilevel models described above. Once again, variables and their interactions with maternal childhood trauma were added one‐by‐one and we assessed whether the added variable significantly improved the model, using the Likelihood‐ratio test. Only variables that significantly improved a multilevel model were retained. All three moderators were tested in the same model and when adding these moderation variables and their interactions to our models, we always entered the main effect of prenatal psychopathology first, followed by the interaction of maternal trauma and prenatal psychopathology. Then, we added prenatal circadian cortisol and finally breastfeeding duration, first adding the main effects of these variables and then their interactions with maternal childhood trauma. Sensitivity analyses revealed that changing the order of entering these potential moderating variables or testing these moderators in separate models did not change the significance of any of our findings.

##### 2.4.3.3 Sex‐Specific Analyses

Given that previous research has consistently found sex differences in HPA axis activity (Panagiotakopoulos and Neigh 2014), we also deviated from our preregistration (in response to a reviewer's request) by carrying out all analyses described above separately for girls and boys. Moreover, we also deviated from our preregistration in response to a reviewer's request by creating a single circadian cortisol multilevel model and a single cortisol reactivity multilevel model, containing data from both the 12 month and the 6‐year measurement, and subsequently carrying out the preregistered analyses, the moderation analyses and the sex‐stratified analyses with these models. These single models contained a time variable indicating the assessment time point (12 months, 6 years), and a second time variable containing the measurement times within each assessment time point.

##### 2.4.3.4 Subtypes of Trauma

Due to the high correlation between maternal childhood maltreatment (CTQ) and maternal childhood peer and family violence (ACE‐IQ) (*r *= 0.59, *p *< 0.001), we also calculated a composite score, by calculating *z*‐scores for all CTQ and ACE items and then calculating a sum score of all items, and assessed its association with children's total circadian cortisol output and cortisol reactivity. Moreover, in line with Colich et al. (2020), who found that early life adversity involving threat was associated with biological aging whereas early life adversity involving deprivation was not, we attempted to use the CTQ and ACE‐IQ subscales to create a threat and an adversity variable. We therefore combined the CTQ subscales emotional neglect and physical neglect to create a deprivation variable, and created a threat variable by combining the CTQ subscales emotional abuse, sexual abuse, and physical abuse, and the ACE‐IQ subscales domestic violence and bullying. Then, we assessed the associations between these threat and deprivation variables and children's total circadian cortisol output and cortisol reactivity.

Additionally, in response to reviewers’ requests to examine whether specific subtypes of maternal childhood trauma were associated with children's HPA axis activity—since previous research has found differential associations between different types of childhood trauma and child HPA axis functioning (Robinson 2016)—we exploratorily carried out the preregistered multilevel analyses using the different subscales of the CTQ rather than the total CTQ score as independent variables. Separate analyses were carried out for the five CTQ subscales.

## 3 Results

### 3.1 Descriptive Statistics and Correlations

Means and standard deviations of all maternal childhood trauma and child cortisol variables can be found in Table 1. Regarding the prevalence of maternal childhood trauma, 4.8% of mothers experienced low to severe physical abuse (0.6% experienced severe abuse), 16.8% experienced low to severe emotional abuse (4.2% experienced severe abuse), 15.6% experienced low to severe sexual abuse (3.0% experienced severe abuse), 36.5% of the sample experienced low to severe emotional neglect (4.2% experienced severe neglect), and 18.6% experienced low to severe physical neglect (1.2% experienced severe neglect). Furthermore, half of the sample (52.4%) did not experience any childhood peer and family violence (ACE‐IQ), 27.1% experienced one ACE, 10.6% experienced two ACEs, 7.6% experienced three ACEs, and 2.4% experienced four ACEs. A total of 32.4% of the sample reported no or only minimal trauma, that is, they reported no experiences of family and peer violence (ACE‐IQ), and also reported no or only minimal experiences of abuse or neglect (CTQ).

**TABLE 1:** Descriptive statistics of maternal childhood maltreatment experiences, maternal family and peer violence experiences, and child circadian cortisol and cortisol reactivity (untransformed).

Variable	*N*	Mean	*SD*	Minimum	Maximum
**Maternal childhood maltreatment (CTQ)**	167	32.99	8.69	25.00	65.63
**Maternal childhood family and peer violence (ACE‐IQ)**	170	0.81	1.06	0.00	4.00
**Circadian cortisol 12 months**					
T1 (after waking up)	133	16.16	5.71	3.60	37.00
T2 (11:00)	136	9.02	4.33	3.60	27.00
T3 (15:00)	134	7.64	4.05	2.10	31.00
T4 (19:00)	136	4.50	3.16	1.40	19.10
**Circadian cortisol 6 years**					
T1 (after waking up)	135	15.20	5.09	4.70	28.50
T2 (11:00)	134	7.36	2.52	1.50	19.75
T3 (15:00)	135	5.33	1.85	1.80	13.50
T4 (19:00)	136	2.39	1.37	1.00	8.80
**Cortisol reactivity 12 months**					
C1 (baseline)	161	7.42	3.37	3.30	22.00
C2 (reactivity)	157	8.02	4.20	2.90	24.00
C3 (reactivity)	157	7.90	3.56	3.10	24.00
C4 (recovery)	133	7.07	3.05	3.10	20.00
**Cortisol reactivity 6 years**					
C1 (baseline)	140	7.03	3.54	1.80	36.00
C2 (baseline)	141	6.36	3.00	1.30	29.00
C3 (reactivity)	144	6.80	3.27	1.20	21.00
C4 (reactivity)	140	7.00	4.13	1.20	26.00
C5 (recovery)	141	5.92	3.11	1.10	26.00
C6 (recovery)	140	5.64	2.66	1.10	17.30
**Prenatal psychopathology**					
Maternal depression (EPDS)	164	5.29	3.93	0	21
Maternal anxiety (STAI)	163	32.18	8.74	20	64
**Prenatal circadian cortisol**					
Maternal cortisol decline	132	6.71	4.53	−2.80	24.00
Maternal evening cortisol	137	9.46	2.86	0.90	22.00
**Breastfeeding duration**	168	5.42	4.32	0	12

At 12 months, 45.9% of the participating children showed a baseline‐to‐peak increase in cortisol concentrations of 15.5% or higher and can therefore be classified as responders to the stress paradigm according to the classification recommendations of Miller et al. (2013). The average baseline‐to‐peak increase at 12 months was 33.63% (SD = 78.35). At 6 years, children showed an average baseline‐to‐peak increase of 20.35% (SD = 49.80) and 33.1% of children could be classified as responders.

**FIGURE 1:**
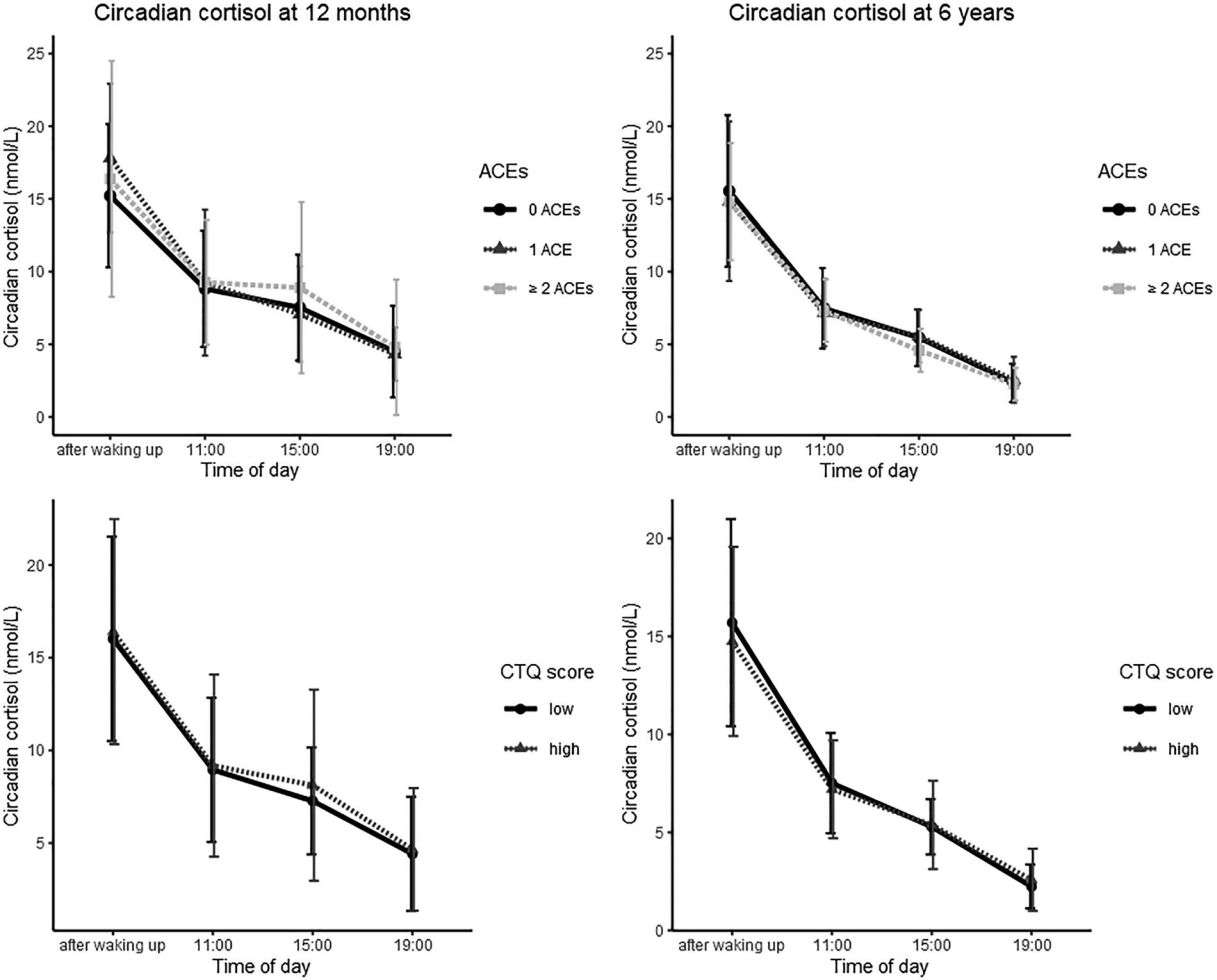
Total circadian cortisol output at 12 months and 6 years. *Note:* The high and low CTQ groups were created using median splits.

**FIGURE 2:**
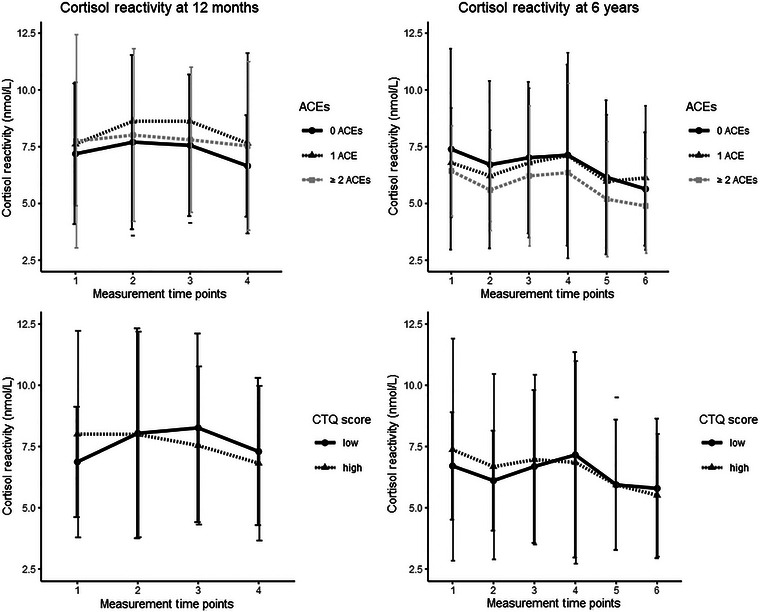
Cortisol reactivity at 12 months and at 6 years. *Note:* The high and low CTQ groups were created using median splits.

For illustrative purposes, we also plotted means and standard deviations of all child cortisol variables (see Figures 1 and 2), separately for children of mothers with low versus high levels of childhood trauma (for the CTQ, low and high trauma groups were created using median splits; for the ACE‐IQ, mothers with 0 ACEs were compared with mothers with 1 ACE and mothers with ≥2 ACEs). While Figure 1 shows almost identical diurnal cortisol patterns for children of mothers with low versus high childhood trauma, cortisol reactivity patterns (Figure 2), however, seem to differ somewhat more between children of mothers with low versus high childhood trauma.

The trauma variables, maternal childhood maltreatment (CTQ) and maternal childhood peer and family violence (ACE‐IQ), correlated strongly with each other (*r* = 0.59, *p* < 0.001). Neither trauma variable correlated with any cortisol variables, nor with the covariates child sex and maternal education. Child sex did not correlate with any trauma or cortisol variables, but maternal education correlated with the second circadian cortisol measure at 12 months (t2; *r* = −0.18, *p* = 0.03), and the third circadian cortisol measure at 6 years (t3; *r* = 0.19, *p *= 0.04). At age six, circadian and reactivity scores correlated strongly with each other (see Table 2), while circadian and reactivity scores did not correlate at age 12 months.

**TABLE 2:** Pearson correlations of all circadian cortisol and cortisol reactivity variables.

	1.	2.	3.	4.	5.	6.	7.	8.	9.	10.	11.	12.	13.	14.	15.	16.	17.	18.
Circadian cortisol 12 months																		
1. T1 (after waking up)	—																	
2. T2 (11:00)	0.21*	—																
3. T3 (15:00)	0.22*	0.20*	—															
4. T4 (19:00)	0.06	0.30^**^	0.35^***^	—														
Circadian cortisol 6 years																		
5. T1 (after waking up)	0.00	0.15	0.19*	0.18	—													
6. T2 (11:00)	0.02	0.15	−0.09	−0.02	0.32^***^	—												
7. T3 (15:00)	−0.09	0.08	−0.03	0.06	0.35^***^	0.32^***^	—											
8. T4 (19:00)	−0.02	−0.11	−0.13	−0.10	0.13	0.31^***^	0.30^***^	—										
Cortisol reactivity 12 months																		
9. C1 (baseline)	−0.07	0.09	0.10	0.12	0.03	−0.08	0.02	0.04	—									
10. C2 (reactivity)	−0.05	−0.05	−0.01	0.05	−0.13	−0.14	−0.04	0.01	0.17*	—								
11. C3 (reactivity)	0.04	0.02	0.02	0.07	−0.14	−0.13	−0.07	−0.01	0.18*	0.82^***^	—							
12. C4 (recovery)	0.01	0.02	−0.04	0.00	−0.09	−0.03	0.03	−0.03	0.12	0.72^***^	0.83^***^	—						
Cortisol reactivity 6 years																		
13. C1 (baseline)	0.03	0.19^*^	0.00	0.00	0.32^***^	0.41^***^	0.33^***^	0.33^***^	−0.08	−0.06	−0.10	−0.09	—					
14. C2 (baseline)	−0.04	0.14	−0.20*	0.02	0.18*	0.35^***^	0.35^***^	0.27^**^	−0.01	0.01	−0.05	−0.05	0.72^***^	—				
15. C3 (reactivity)	−0.05	0.01	−0.17	0.06	0.13	0.25^**^	0.22^*^	0.18^*^	−0.01	0.13	0.03	0.00	0.52^***^	0.79^***^	—			
16. C4 (reactivity)	−0.06	−0.05	−0.12	0.04	0.06	0.27^**^	0.15	0.18^*^	−0.03	0.23^**^	0.14	0.04	0.48^***^	0.71^***^	0.96^***^	—		
17. C5 (recovery)	−0.06	−0.06	−0.14	0.04	0.10	0.32***	0.23**	0.30**	0.03	0.20*	0.10	0.03	0.47^***^	0.68^***^	0.89^***^	0.94^***^	—	
18. C6 (recovery)	−0.08	−0.10	−0.11	0.05	0.04	0.25^**^	0.17	0.26^**^	0.04	0.28^**^	0.17	0.06	0.46^***^	0.66^***^	0.84^***^	0.90^***^	0.97^***^	—

* *p *< 0.05.

** *p *< 0.01.

*** *p* < 0.001.

### 3.2 Associations between Maternal Childhood Trauma and Cortisol Reactivity

Table 3 shows the best fitting mixed effects models for cortisol reactivity at age 12 months, 6 years, and across both time points. In all three models, quadratic time was significantly associated with cortisol reactivity, indicating that the cortisol stress response was indeed described better by a quadratic rather than a linear model. In the overall model containing cortisol reactivity measures from both the 12 month and the 6 year measurement, cortisol concentrations were higher at the 12 month measurement compared with the 6 year measurement (*F*(1, 123.80) = 8.66, *p *< 0.001, *B *= −0.99, 95% CI [−0.32; −1.65]). In our preregistered models for the ages 12 months and 6 years, maternal childhood maltreatment was not significantly associated with cortisol reactivity. Maternal childhood peer and family violence was not included in any of the multilevel models, because adding this variable did not significantly improve model fit. These results remained the same when exploratorily combining the 12 month and 6‐year cortisol reactivity measures into a single multilevel model.

**TABLE 3:** Estimates for the best fitting mixed effects model for cortisol reactivity at 12 months and 6 years.

Cortisol reactivity at age 12 months				Cortisol reactivity at age 6 years				Cortisol reactivity across ages			
	Estimate	SE	*p*		Estimate	SE	*p*		Estimate	SD	*p*
Full sample				Full sample				Full sample			
Intercept	5.80	0.58	**<0.001**	Intercept	6.72	0.36	**<0.001**	Intercept	6.59	0.27	**<0.001**
								Age child	−0.99	0.34	**<0.01**
Time	1.76	0.52	**<0.001**	Time	0.32	0.16	**0.046**	Measurement time	0.03	0.01	**<0.001**
Time^2^	−0.36	0.10	**<0.001**	Time^2^	−0.08	0.02	**<0.001**	Measurement time^2^	−0.00	0.00	**<0.001**
Maternal education	−0.03	0.17	0.84	Maternal education	0.12	0.19	0.54	Maternal education	−0.01	0.13	0.95
Maternal childhood maltreatment (CTQ)	−0.00	0.03	0.91	Maternal childhood maltreatment (CTQ)	−0.00	0.03	0.89	Maternal childhood maltreatment (CTQ)	−0.01	0.02	0.65
Psychopathology	−0.07	0.26	0.80					Psychopathology	0.00	0.20	0.99
				Breastfeeding	0.03	0.06	0.63	Breastfeeding	0.01	0.04	0.83
Prenatal cortisol slope Deviance	0.03 2296.67	0.05	0.63	Prenatal cortisol slope Deviance	−0.00 2739.56	0.06	0.99	Prenatal cortisol slope Deviance	0.00 5189.18	0.04	0.95
Girls only				Girls only				Girls only			
Intercept	5.95	0.82	**<0.001**	Intercept	7.07	0.53	**<0.001**	Intercept	7.04	0.40	**<0.001**
								Age child	−0.48	0.49	0.33
Time	1.43	0.74	0.05	Time	0.43	0.28	0.13	Measurement time	0.03 −0.00	0.01	**0.03**
Time^2^	−0.29	0.15	0.05	Time^2^	−0.11	0.04	**<0.01**	Measurement time^2^		0.00	**<0.001**
Maternal education	−0.04	0.28	0.89	Maternal education	−0.46	0.29	0.12	Maternal education	−0.28	0.22	0.21
				Breastfeeding	0.23	0.09	**0.02**	Breastfeeding	0.06	0.07	0.40
Prenatal cortisol slope	0.08	0.08	0.32	Prenatal cortisol slope	−0.08	0.09	0.36	Prenatal cortisol slope	0.00	0.06	0.96
Deviance	967.26			Deviance	1334.00			Deviance	2360.26		
Boys only				Boys only				Boys only			
Intercept	5.66	0.82	**<0.001**	Intercept	6.53	0.48	**<0.001**	Intercept	6.22	0.38	**<0.001**
								Age child	−1.40	0.45	**<0.01**
Time	2.03	0.74	**<0.01**	Time	0.21	0.17	0.23	Measurement time	0.04	0.01	**<0.01**
Time^2^	−0.41	0.15	**<0.01**	Time^2^	−0.06	0.02	**0.01**	Measurement time^2^	−0.00	0.00	**<0.001**
Maternal education	−0.06	0.22	0.79	Maternal education	0.44	0.24	0.07	Maternal education	0.14	0.18	0.42
Maternal childhood maltreatment (CTQ)	−0.01	0.04	0.80	Maternal childhood maltreatment (CTQ)	−0.03	0.05	0.51	Maternal childhood maltreatment (CTQ)	−0.02	0.03	0.65
Psychopathology	0.03	0.35	0.93					Psychopathology	−0.12	0.28	0.68
Prenatal cortisol slope	−0.01	0.07	0.88	Prenatal cortisol slope	0.06	0.08	0.48	Prenatal cortisol slope	0.00	0.06	0.96
Deviance	1328.29			Deviance	1352.15			Deviance	2842.42		

Note:  Signficant values are bolded.

Exploratory sensitivity analyses, consisting of simple multiple regression analyses, led to the same results: maternal childhood maltreatment was not associated with children's highest poststressor concentrations (adjusted for baseline cortisol concentrations) at 12 months or at 6 years. Similarly, exploratory analyses for cortisol reactivity at 6 years, which only used the lowest baseline, highest peak, and lowest recovery measure, did not lead to different results and the association between maternal childhood trauma and child cortisol reactivity at age 6 years remained nonsignificant.

When exploratorily adding the maternal psychopathology, prenatal circadian cortisol slope, and breastfeeding duration as moderators to the multilevel models, maternal childhood maltreatment did not become a significant predictor of child cortisol reactivity either and neither were any of these moderator variables directly significantly related to children's cortisol reactivity. Maternal evening cortisol and the interaction terms between the moderator variables and maternal childhood trauma were not included in the best‐fitting models depicted in Table 4, as none of these interaction terms significantly improved model fit.

**TABLE 4:** Estimates for the best fitting mixed effects model for the total circadian cortisol output at 12 months and 6 years.

Total circadian cortisol output at 12 months				Total circadian cortisol output at 6 years				Total circadian cortisol output across ages			
	Estimate	SE	*p*		Estimate	SE	*p*		Estimate	SD	*p*
Full sample Intercept	22.72	0.66	**<0.001**	Full sample Intercept	21.86	0.52	**<0.001**	Full sample Intercept	21.53	0.44	**<0.001**
								Age child	−1.63	0.28	**<0.001**
Time of day	−0.00	0.00	**<0.001**	Time of day	−0.00	0.00	**<0.001**	Time of day	−0.00	0.00	**<0.001**
Maternal education	−0.18	0.19	0.33	Maternal education	0.30	0.15	**0.046**	Maternal education	0.00	0.12	0.97
Maternal childhood maltreatment (CTQ)	0.01	0.03	0.72	Maternal childhood maltreatment (CTQ)	−0.01	0.02	0.51	Maternal childhood maltreatment (CTQ)	−0.00	0.02	0.96
Psychopathology	0.29	0.27	0.29					Psychopathology	0.32	0.19	0.09
								CTQ × psychopathology	0.06	0.03	**0.03**
				Breastfeeding	0.01	0.05	0.79	Breastfeeding	0.01	0.04	0.84
Prenatal cortisol slope	0.06	0.05	0.29	Prenatal cortisol slope	0.14	0.04	**<0.01**	Prenatal cortisol slope	0.09	0.04	**0.01**
								CTQ × prenatal cortisol slope	−0.01	0.01	**0.03**
Deviance	2277.66			Deviance	2239.35			Deviance	4512.44		
Girls only Intercept	23.23	1.04	**<0.001**	Girls only Intercept	22.28	0.80	**<0.001**	Girls only Intercept	21.93	0.67	**<0.001**
								Age child	−1.74	0.40	**<0.001**
Time	−0.00	0.00	**<0.001**	Time	−0.00	0.00	**<0.001**	Measurement time	−0.00	0.00	**<0.001**
Maternal education	0.19	0.32	0.56	Maternal education	0.50	0.23	**0.04**	Maternal education	0.35	0.21	0.11
				Breastfeeding	0.06	0.07	0.39	Breastfeeding	0.08	0.06	0.23
Prenatal cortisol slope	0.03	0.09	0.71	Prenatal cortisol slope	0.13	0.06	**0.04**	Prenatal cortisol slope	0.08	0.06	0.19
Deviance	968.57			Deviance	1063.50			Deviance	2015.18		
Boys only Intercept	22.22	0.85	**<0.001**	Boys only Intercept	21.51	0.68	**<0.001**	Boys only Intercept	21.17	0.58	**<0.001**
								Age child	−1.52	0.35	**<0.001**
Time	−0.00	0.00	**<0.001**	Time	−0.00	0.00	**<0.001**	Measurement time	−0.00	0.00	**<0.001**
Maternal education	−0.29	0.23	0.22	Maternal education	0.14	0.20	0.49	Maternal education	−0.15	0.15	0.32
Maternal ACEs	1.04	0.38	**<0.01**								
Maternal childhood maltreatment (CTQ)	−0.04	0.05	0.41	Maternal childhood maltreatment (CTQ)	0.01	0.04	0.89	Maternal childhood maltreatment (CTQ)	0.02	0.03	0.43
Psychopathology	0.33	0.35	0.35					Psychopathology	0.39	0.23	0.09
Prenatal cortisol slope	0.09	0.07	0.19	Prenatal cortisol slope	0.11	0.07	0.09	Prenatal cortisol slope	0.08	0.04	0.07
Deviance	1318.34			Deviance	1193.32			Deviance	2508.36		

Note:  Signficant values are bolded.

Finally, sex‐stratified analyses revealed that maternal childhood maltreatment was also not significantly related to children's cortisol reactivity when examining only boys or only girls. Notably, while maternal childhood maltreatment was included in the final models for boys as a nonsignificant predictor that improved model fit, it was not included in any of the best‐fitting models for girls, as it did not significantly improve model fit in any of the girls only‐models.

### 3.3 Associations between Maternal Childhood Trauma and Total Circadian Cortisol Output

Table 4 shows the best fitting mixed effects models for circadian cortisol measures at 12 months and 6 years. In both models, time was significantly associated with total circadian cortisol output (*p*’s < 0.001), indicating that cortisol concentrations decline over the day. In the exploratory combined model of both the 12 month and the 6 year measurements, circadian cortisol output was higher at 12 months than at 6 years of age (*F*(1, 113.46) = 32.82, *p *< 0.001, *B *= −1.63, 95% CI [−1.07; −2.20]). In our preregistered models, maternal childhood maltreatment was once again not significantly associated with the total circadian cortisol output measures at both ages, and maternal childhood peer and family violence was not included in the models, as including this variable did not significantly improve model fit. In the exploratory combined model of circadian cortisol across ages, we did not find a direct significant effect of maternal childhood maltreatment on children's total circadian cortisol output either.

Exploratory sensitivity analyses, consisting of multiple regression analyses, led to the same results: maternal childhood maltreatment was not associated with children's AUCg or slope, neither at 12 months nor at 6 years.

When exploratorily adding the variables prenatal psychopathology, prenatal circadian cortisol, and breastfeeding duration as moderators to our mixed effects models, the direct effect of maternal childhood maltreatment on child total circadian cortisol output remained nonsignificant. At 6 years and in the combined model of both ages, the prenatal circadian cortisol slope was significantly associated with children's total circadian cortisol output (6 years: (*F*(1, 101.46) = 9.27, *p *< 0.01, *B *= 0.14, 95% CI [0.05; 0.23]); combined model: (*F*(1, 111.32) = 6.67, *p *= 0.01, *B *= 0.09, 95% CI [0.02; 0.16]). Moreover, in the combined model, the interaction of maternal childhood maltreatment and prenatal circadian cortisol slope was significantly associated with children's total circadian cortisol output (*F*(1, 124.37) = 5.10, *p *= 0.03, *B *= −0.01, 95% CI [−0.02; −0.001]). Simple slopes analyses revealed that in children of mothers with smaller slopes (i.e., flatter day curves), more maternal maltreatment experiences were associated with higher total circadian cortisol output (*F*(1, 116.12) = 3.33, *p *= 0.07, *B *= 0.05, 95% CI [−0.004; 0.10]), while in children of mothers with larger diurnal cortisol declines, more maternal maltreatment experiences were associated with a lower total circadian cortisol output (*F*(1, 129.54) = 2.58, *p *= 0.11, *B *= −0.05, 95% CI [−0.12; 0.01]).

Additionally, the interaction of maternal childhood maltreatment and prenatal psychopathology was significantly associated with children's total circadian cortisol output in the combined multilevel model (*F*(1, 130.92) = 5.06, *p *= 0.03, *B *= 0.06, 95% CI [0.01; 0.12]). In children of mothers who experienced low levels of prenatal psychopathology, more severe maternal maltreatment experiences were associated with a significantly lower total circadian cortisol output (*F*(1, 123.84) = 4.26, *p *= 0.04, *B *= −0.06, 95% CI [−0.12; −0.002]). In children of mothers who had experienced high levels of prenatal psychopathology, however, more severe maltreatment experiences were associated with a higher total circadian cortisol output (*F*(1, 130.73) = 2.57, *p* = 0.11, *B *= 0.06, 95% CI [−0.02; 0.13]).

When carrying out exploratory sex‐stratified analyses, neither maternal childhood maltreatment nor the interactions between maternal childhood maltreatment and any of the examined moderators were significant predictors of girls’ or boys’ total circadian cortisol output. However, in boys only, more experiences of maternal childhood family and peer violence were associated with a higher total circadian cortisol output at 12 months of age (*F*(1, 50.00) = 7.61, *p *< 0.01, *B *= 1.04, 95% CI [0.28; 1.80]).

### 3.4 Exploratory Analyses: Associations between Different Subtypes of Maternal Childhood Trauma and Child HPA Axis Function

A composite maternal childhood trauma score, combining both the CTQ and the ACE‐IQ, was not significantly associated with any child cortisol measures. Maternal childhood experiences of threat or of deprivation were not significantly associated with any child cortisol measures either.

Finally, none of the subtypes of maternal childhood maltreatment—physical abuse, sexual abuse, emotional abuse, physical neglect, and emotional neglect—were significantly associated with children's total circadian cortisol output or cortisol reactivity concentrations at 12 months, 6 years, or in the combined multilevel model (see Table S1 in the Supporting Information for the best‐fitting models of the different maltreatment subtypes for all total circadian cortisol output and cortisol reactivity models).

## 4 Discussion

This study investigated associations between maternal childhood trauma experiences and cortisol reactivity and total circadian cortisol output, measured when children were 12 months and 6 years old. Contrary to our hypotheses, neither maternal childhood maltreatment nor maternal childhood experiences of family and peer violence were directly associated with child cortisol reactivity or total circadian cortisol output at 12 months or at 6 years of age, or when examining children's total circadian cortisol output and cortisol reactivity across both measurement time points. When carrying out exploratory sex‐stratified analyses, maternal experiences of family and peer violence emerged as a significant predictor for boys’ but not girls’ total circadian cortisol output at 12 months only. Exploratory moderation analyses revealed that prenatal circadian cortisol slope and prenatal psychopathology significantly moderated the association between maternal childhood maltreatment experiences and children's total circadian cortisol output, when combining cortisol measurements from 12 months and 6 years of age in one model.

Our nonsignificant findings regarding the direct associations between maternal childhood trauma and child cortisol reactivity are in line with and extend studies by Brand et al. (2010), Lindrose (2021), and Schlueter (2017), who also found no such associations in 4–44‐month‐olds, by replicating these findings in 12‐month and 6‐year‐old children. Our largely nonsignificant findings regarding the direct associations between maternal childhood trauma and child total circadian cortisol output are in line with Schlueter (2017) and with Fuchs et al. (2016). At the same time, our findings contradict other theoretical (Yehuda and Lehrner 2018) and empirical evidence suggesting that maternal childhood trauma may impact child HPA axis functioning and may be associated specifically with blunted cortisol reactivity in children (Khoury et al. 2021; Parade et al. 2019; Robinson 2016), and lower baseline cortisol concentrations (Brand et al. 2010; Khoury et al. 2021). Notably, these inconsistent results in the previous literature do not appear to depend on the prevalence of trauma in the studies’ samples, as this prevalence varied between 30 and 46.6% in studies finding no association (Brand et al. 2010; Fuchs et al. 2016) and between 29 and 51% in studies finding significant associations (Brand et al. 2010; Khoury et al. 2021; Parade et al. 2019; Robinson 2016). In sum, research on the potential role of the HPA axis in the intergenerational transmission of the effects of childhood trauma thus remains rather inconsistent. There are multiple factors which might explain these inconsistent findings. These factors will be discussed one by one in the following paragraphs.

First, the association between maternal childhood trauma experiences and child HPA axis functioning might be moderated by other factors and might thus only exist in certain mother–child groups. This is in line with some of the results of our exploratory moderation analyses, which suggested that maternal childhood maltreatment experiences were only associated with higher total circadian cortisol output—which can be a risk factor for adverse developmental outcomes such as stress‐related illnesses, when cortisol concentrations are chronically elevated (Gunnar and Quevedo 2007)—when mothers experienced higher levels of prenatal psychopathology or more prenatal circadian cortisol dysregulation. Similarly, Thomas et al. (2018) only found an indirect effect between maternal ACEs and offspring cortisol reactivity, which was mediated by increased maternal cortisol activity during pregnancy. Maternal stress during pregnancy could thus play a crucial role in determining whether and how maternal childhood trauma experiences affect the next generation's HPA axis functioning.

Since maternal childhood trauma could also affect children's HPA axis functioning postnatally (Yehuda and Lehrner 2018), for example by affecting the environment children grow up in, it seems plausible that postnatal factors could moderate this association as well. One postnatal pathway might involve breastfeeding duration, since mothers can continue sending physiological signals to their child through the constituents within their breast milk, which in turn could affect child HPA axis development (de Weerth et al. 2022). Although one could therefore hypothesize that the link between maternal childhood trauma and child HPA axis functioning might depend on whether and for how long mothers breastfed their children, we found no evidence for such a moderating effect. A potential explanation for these findings could be that rather than quantity, that is, duration of breastfeeding, quality of breast milk, that is, the specific constituents within the mother's breast milk, might play a larger role.

Furthermore, nonbiological factors, such as maternal parenting might be relevant postnatal moderating factors. On the one hand, mothers who use suboptimal parenting strategies might thereby contribute to creating a more unpredictable, stressful environment for their children to grow up in (R. Roberts et al. 2004), which their children's stress systems might adapt to as children grow older (Yehuda and Lehrner 2018). Conversely, mothers who are able to engage in sensitive, positive parenting strategies, despite their own trauma history, might be able to mitigate the effects of their own traumatic experiences on their children through their parenting (Gunnar and Fisher 2006; Sciaraffa, Zeanah, and Zeanah 2018). Consequently, in these cases maternal childhood trauma would not be associated with children's HPA axis activity. In line with this, Hertz (2019) found maternal dispositional mindfulness, which is thought to relate to positive parenting and secure attachment (Duncan, Coatsworth, and Greenberg 2009; Goodall, Trejnowska, and Darling 2012), to moderate the association between maternal childhood maltreatment and offspring cortisol reactivity, with maternal child maltreatment being related to better child cortisol recovery (i.e., a quicker decrease in cortisol concentrations following exposure to a stressor) only in mothers high in mindfulness.

Additionally, the association between maternal childhood trauma and child HPA axis might depend on certain child characteristics, such as child sex, given that previous research has consistently found sex differences in HPA axis functioning (Panagiotakopoulos and Neigh 2014). In line with this, we found a significant association between more experiences of childhood family and peer violence in mothers and a higher total circadian cortisol output at 12 months of age only in boys. Importantly, these sex‐stratified analyses were exploratory and thus require further replication. Nevertheless, these findings do indicate that exploring the role of child sex in the association between maternal childhood trauma and child HPA axis functioning might be a worthwhile avenue for future research. In sum, future research should focus on further examining the potential moderating effects of various risk and protective factors—both biological and nonbiological as well as prenatal and postnatal factors—on the association between maternal childhood trauma and child HPA axis activity.

Alternatively, while we did find some associations between maternal childhood trauma and child total circadian cortisol output in combination with other risk factors during pregnancy, the prevalence of maternal childhood trauma in our community sample might have been too low to detect a direct association with child HPA axis activity. While a rather high percentage of our sample reported experiences of low to severe emotional neglect (36.5%), the prevalence rates for sexual abuse (15.6% experienced low to severe abuse, 3.0% severe abuse) and physical abuse (4.8% experienced low to severe abuse, 0.6% severe abuse) were much lower. It could be possible that the association between maternal childhood trauma and child HPA axis activity might only be detectable in samples with higher trauma exposure. Given the rather low prevalence of severe maltreatment experiences in our own sample in combination with our largely nonsignificant results, it also seems plausible that only severe maternal trauma exposure and/or trauma exposure in combination with other risk factors, such as maternal psychopathology, might affect children's HPA axis development.

Finally, factors related to the type and timing of mothers’ traumatic childhood experiences might have led to the mixed findings in previous studies. In the current study, we found no evidence supporting the hypothesis that different types of childhood maltreatment might be differentially associated with children's HPA axis functioning. This is in line with previous findings indicating that different types of maltreatment frequently co‐occur (Kim et al. 2017), making it difficult to clearly separate the effects of different maltreatment subtypes from each other. Our findings contradict previous research by Robinson (2016), however, who found a significant association between maternal childhood trauma and child cortisol reactivity only for sexual abuse, but not for physical abuse. Notably, however, we found some evidence for associations between maternal maltreatment (measured with the CTQ) and psychopathology or maternal circadian cortisol slope, but almost no effects for maternal experiences of peer and family violence (measured with items from the ACE‐IQ). These findings could suggest that maltreatment experiences may be more strongly associated with children's HPA axis development than experiences of family and/or peer violence.

Similarly, the exact timing of trauma exposure appears to be an important modulating factor of the biological effect of childhood trauma, particularly regarding the HPA axis (Agorastos et al. 2019; Bosch et al. 2012). Bosch et al. (2012) found, for example, that adversity experienced during child ages 6–11 years was associated with high salivary cortisol concentrations when those children were exposed to a stress test, whereas adversities during the ages 12–15 years were associated with low cortisol concentrations. If childhood trauma can thus lead to different biological effects in trauma survivors themselves, depending on the timing of the experienced trauma, it is plausible that this timing may also affect the biological effects of childhood trauma on the next generation. Future research should therefore further examine the effects of the type and particularly the timing of mothers’ childhood experiences on their children's HPA axis functioning.

Although we found some evidence for an association between maternal childhood maltreatment experiences and child total circadian cortisol output in combination with other maternal risk factors, specifically prenatal circadian cortisol slope and psychopathology, we found no evidence for direct or indirect associations between maternal childhood trauma and child cortisol reactivity. This might partially be due to the specific moderators we examined: we only assessed prenatal circadian cortisol, not maternal cortisol reactivity. It could be the case that maternal cortisol reactivity, rather than the circadian cortisol slope, might play a more important role in relation to child circadian cortisol output. Another reason for the lack of findings concerning child cortisol reactivity could be that while we did observe significant changes in cortisol concentrations in response to the SSP at 12 months and the CREST at 6 years, the observed cortisol increases were relatively mild. The average cortisol increase of 20.35% in response to the CREST is lower than what was observed in previous studies using the CREST (de Weerth et al. 2013) or the somewhat similar Trier Social Stress Test for children (Kudielka et al. 2004; Yim et al. 2010). The relatively mild stress response to the SSP at 12 months is in line with the mixed findings in the previous literature, in which some studies observed increases in cortisol concentrations (Dismukes et al. 2018; Hertsgaard et al. 1995), while other studies did not (DePasquale et al. 2018; Dozier et al. 2008). In general, it is not uncommon for stress tests to not elicit cortisol increases in every participant (de Weerth et al. 2013; Gunnar, Talge, and Herrera 2009; Klimes–Dougan et al. 2001), particularly because stress tests for children, like the CREST, are carefully designed to be stressful, but not too stressful (de Weerth et al. 2013). In the current study, this may have resulted in the stress tasks—particularly the CREST—not being stressful enough to elicit a cortisol increase in the majority of the participants. Another reason for the lack of findings concerning child cortisol reactivity at the 12 month measurement could be that children appear to enter a period of cortisol hyporesponsivity during the second half of their first year of life, during which it may be difficult to elicit a cortisol increase in children (Gunnar and Donzella 2002). In the current study, however, the percentage of children exhibiting a noticeable increase in cortisol concentrations in response to the stress paradigm they were exposed to was higher at 12 months (45.9% of children classified as responders) than at 6 years (33.1% of children classified as responders), thus casting doubt on whether our lack of significant associations is truly due to this hyporesponsive period. Further research concurrently examining associations between maternal childhood trauma and child circadian cortisol output and cortisol reactivity is therefore needed to investigate whether and why these two aspects of HPA axis functioning might be differentially associated with maternal childhood trauma.

This study has a number of strengths: we measured both cortisol reactivity and circadian cortisol output and measured both in infancy and middle childhood. This makes our study one of the first to investigate the association between maternal childhood trauma and child HPA axis functioning in older, school‐aged children and across different stages of child development. We also comprehensively assessed childhood trauma by measuring both abuse and neglect (using the CTQ) and peer and family violence (using the ACE‐IQ) and we preregistered our data analyses. Nevertheless, this study also has limitations. Our study sample consisted predominantly of well‐educated mothers, thus limiting the generalizability of our findings to the general population. Moreover, maternal childhood trauma was assessed retrospectively when the mothers’ children were 8 years old and thus after children's cortisol concentrations were assessed at 12 months and 6 years. However, since retrospective assessments of childhood trauma have been found to be stable over time (Goltermann et al. 2021), our results would likely not be substantially different had we measured maternal childhood trauma at an earlier time point. Also, our modest sample size in combination with the relatively low prevalence of maternal childhood trauma in our community sample meant that we might have had insufficient statistical power to uncover more modest effects, which could have become statistically significant in larger samples (such as the small differences visible in Figure 2). Future research should therefore utilize larger sample sizes, to be able to uncover smaller associations between maternal childhood maltreatment and child HPA axis functioning. Finally, the current study focused on examining moderating effects of maternal prenatal psychopathology and maternal prenatal circadian cortisol slope. However, these factors could also play a mediating role in the intergenerational association between maternal childhood trauma and child HPA axis functioning (Barclay et al. 2023; Buss et al. 2017). While our moderate sample size did not allow us to do so properly, future studies utilizing larger sample sizes should explore these potential mediating effects further.

In conclusion, within this community sample, we largely found no evidence for a direct association between maternal childhood trauma experiences and child cortisol reactivity or circadian cortisol output, neither at 12 months nor at 6 years. However, we did find interaction effects with maternal childhood trauma and both prenatal psychopathology and prenatal circadian cortisol slope in predicting child total circadian cortisol output. To the best of our knowledge, this is one of the first studies examining the relations of maternal childhood trauma experiences with school‐aged children's HPA axis functioning and the associations between maternal childhood trauma and child circadian cortisol. Further research is needed to replicate our findings in larger samples and to study school‐aged and older children and high‐risk populations. Furthermore, future research should further investigate moderators and mediators of the potential association between maternal childhood trauma and offspring HPA axis functioning. Next to prenatal psychopathology and HPA axis functioning, potential moderators could include other biological factors, such as the constituents of mothers’ breast milk, immune system factors (De Bellis and Zisk 2014), or gut microbiota (Y. Zhang et al. 2022); but also nonbiological factors, such as mothers’ caregiving behavior (Penner et al. 2023) or the degree to which mothers have resolved their traumatic experiences (Iyengar et al. 2019).

## Ethics Statement

This study was part of an ongoing longitudinal project on children's psychobiological development (BIBO study; Basal Influences on Child Development), which received ethical approval from the Ethics Committee Social Sciences of the Radboud University (ECG/AvdK/07.563).

## Conflicts of Interest

The authors declare no conflicts of interest.

## Supporting information




**Supplementary Material—*Table 1*** Best‐fitting mixed effects models of the different subtypes of maternal childhood maltreatment predicting children's cortisol concentrations.

## Data Availability

The data that support the findings of this study are available from the corresponding author upon reasonable request.
