# Supplementary material for: Maternal Childhood Trauma and Offspring Hypothalamic–Pituitary–Adrenal Axis Function from Infancy to 6 Years of Age
Source: Dev Psychobiol. 2025 Feb 17;67(2):e70029. doi: 10.1002/dev.70029 (PMC11831242; doi:10.1002/dev.70029)
Supplement: Supplementary file 1 — Supplementary Material—Table 1 Best‐fitting mixed effects models of the different subtypes of maternal childhood maltreatment predicting children's cortisol concentrations. [file DEV-67-e70029-s001.docx]

**Supplementary Materials**

| *Table 1.*  Best-fitting mixed effects models of the different subtypes of maternal childhood maltreatment predicting children’s cortisol concentrations | | | | | | | | | | | | | | | | | | |
| --- | --- | --- | --- | --- | --- | --- | --- | --- | --- | --- | --- | --- | --- | --- | --- | --- | --- | --- |
|  | Circadian cortisol at 12 months | | | Circadian cortisol at 6 years | | | Circadian cortisol across ages | | | Cortisol reactivity at 12 months | | | Cortisol reactivity at 6 years | | | Cortisol reactivity across ages | | |
|  | Estimate | *SE* | *p* | Estimate | *SD* | *p* | Estimate | *SD* | *p* | Estimate | *SD* | *p* | Estimate | *SD* | *p* | Estimate | *SD* | *p* |
| **Child physical abuse** |  |  |  |  |  |  |  |  |  |  |  |  |  |  |  |  |  |  |
| Intercept | 22.70 | 0.65 | **< .001** | 21.87 | 0.52 | **< .001** | 21.50 | 0.44 | **< .001** | 5.80 | 0.58 | **< .001** | 6.73 | 0.36 | **< .001** | 6.22 | 0.38 | **< .001** |
| Age child |  |  |  |  |  |  | 1.58 | 0.28 | **< .001** |  |  |  |  |  |  | 1.40 | 0.45 | **< .01** |
| Measurement time | -0.00 | 0.00 | **< .001** | -0.00 | 0.00 | **< .001** | -0.00 | 0.00 | **< .001** | 1.77 | 0.52 | **< .001** | 0.32 | 0.16 | **.046** | 0.04 | 0.01 | **< .001** |
| Measurement time squared |  |  |  |  |  |  |  |  |  | -0.40 | 0.10 | **< .001** | -0.08 | 0.02 | **< .001** | -0.00 | 0.00 | **< .001** |
| Maternal education | -0.20 | 0.19 | .28 | 0.30 | 0.15 | **.047** | 0.07 | 0.12 | .59 | -0.04 | 0.17 | .81 | 0.12 | 0.19 | .53 | 0.15 | 0.18 | .40 |
| Physical abuse | -0.23 | 0.30 | .45 | -0.03 | 0.23 | .88 | -0.12 | 0.20 | .54 | -0.22 | 0.27 | .42 | 0.27 | 0.34 | .43 | -0.06 | 0.52 | .90 |
| Psychopathology | 0.27 | 0.28 | .32 |  |  |  | 0.18 | 0.18 | .34 | -0.07 | 0.26 | .78 |  |  |  | -0.13 | 0.28 | .64 |
| Breastfeeding |  |  |  | 0.01 | 0.05 | .77 | -0.00 | 0.04 | .96 |  |  |  | 0.03 | 0.06 | .68 |  |  |  |
| Maternal cortisol slope | 0.06 | 0.05 | .25 | 0.14 | 0.05 | **< .01** | 0.10 | 0.04 | **< .01** | 0.03 | 0.05 | .60 | -0.00 | 0.06 | .95 | 0.00 | 0.06 | .95 |
|  |  |  |  |  |  |  |  |  |  |  |  |  |  |  |  |  |  |  |
| Deviance | 2272.62 |  |  | 2235.23 |  |  | 4504.94 |  |  | 2291.53 |  |  | 2734.31 |  |  | 2837.25 |  |  |
| **Child sexual abuse** |  |  |  |  |  |  |  |  |  |  |  |  |  |  |  |  |  |  |
| Intercept | 22.70 | 0.65 | **< .001** | 21.87 | 0.52 | **< .001** | 21.50 | 0.44 | **< .001** | 5.80 | 0.58 | **< .001** | 6.71 | 0.36 | **< .001** | 6.22 | 0.37 | **< .001** |
| Age child |  |  |  |  |  |  | 1.59 | 0.28 | **< .001** |  |  |  |  |  |  | 1.41 | 0.45 | **< .01** |
| Measurement time | -0.00 | 0.00 | **< .001** | -0.00 | 0.00 | **< .001** | -0.00 | 0.00 | **< .001** | 1.76 | 0.52 | **< .001** | 0.32 | 0.16 | **.046** | 0.04 | 0.01 | **< .001** |
| Measurement time squared |  |  |  |  |  |  |  |  |  | -0.36 | 0.10 | **< .001** | -0.08 | 0.02 | **< .001** | -0.00 | 0.00 | **< .001** |
| Maternal education | -0.21 | 0.19 | .26 | 0.30 | 0.15 | **.04** | 0.06 | 0.12 | .61 | -0.03 | 0.17 | .87 | 0.12 | 0.19 | .53 | 0.14 | 0.18 | .42 |
| Sexual abuse | -0.17 | 0.16 | .30 | -0.09 | 0.16 | .57 | -0.13 | 0.12 | .28 | 0.05 | 0.17 | .77 | -0.11 | 0.25 | .65 | -0.14 | 0.21 | .51 |
| Psychopathology | 0.32 | 0.27 | .24 |  |  |  | 0.20 | 0.18 | .28 | -0.08 | 0.26 | .77 |  |  |  | -0.09 | 0.28 | .74 |
| Breastfeeding |  |  |  | 0.02 | 0.05 | .71 | 0.00 | 0.04 | .93 |  |  |  | 0.04 | 0.06 | .57 |  |  |  |
| Maternal cortisol slope | 0.06 | 0.05 | .26 | 0.13 | 0.05 | **< .01** | 0.10 | 0.04 | **< .01** | 0.02 | 0.05 | .64 | -0.00 | 0.06 | .97 | 0.00 | 0.06 | .96 |
|  |  |  |  |  |  |  |  |  |  |  |  |  |  |  |  |  |  |  |
| Deviance | 2273.28 |  |  | 2235.67 |  |  | 4505.09 |  |  | 2292.99 |  |  | 2735.35 |  |  | 2838.58 |  |  |
| **Child emotional abuse** |  |  |  |  |  |  |  |  |  |  |  |  |  |  |  |  |  |  |
| Intercept | 22.73 | 0.66 | **< .001** | 21.87 | 0.52 | **< .001** | 21.51 | 0.44 | **< .001** | 5.80 | 0.58 | **< .001** | 6.72 | 0.36 | **< .001** | 6.22 | 0.38 | **< .001** |
| Age child |  |  |  |  |  |  | 1.59 | 0.29 | **< .001** |  |  |  |  |  |  | 1.40 | 0.45 | **< .01** |
| Measurement time | -0.00 | 0.00 | **< .001** | -0.00 | 0.00 | **< .001** | -0.00 | 0.00 | **< .001** | 1.77 | 0.52 | **< .001** | 0.32 | 0.16 | **.046** | 0.04 | 0.01 | **< .001** |
| Measurement time squared |  |  |  |  |  |  |  |  |  | -0.36 | 0.10 | **< .001** | -0.08 | 0.02 | **< .001** | -0.00 | 0.00 | **< .001** |
| Maternal education | -0.17 | 0.19 | .36 | 0.30 | 0.15 | **.04** | 0.08 | 0.12 | .51 | -0.03 | 0.17 | .84 | 0.11 | 0.19 | .58 | 0.14 | 0.18 | .43 |
| Emotional abuse | 0.06 | 0.09 | .50 | 0.03 | 0.07 | .71 | 0.04 | 0.06 | .52 | -0.00 | 0.09 | .96 | -0.06 | 0.10 | .51 | -0.04 | 0.10 | .72 |
| Psychopathology | 0.27 | 0.28 | .33 |  |  |  | 0.17 | 0.19 | .37 | -0.06 | 0.26 | .80 |  |  |  | -0.10 | 0.28 | .72 |
| Breastfeeding |  |  |  | 0.01 | 0.05 | .78 | -0.00 | 0.04 | .90 |  |  |  | 0.03 | 0.06 | 0.59 |  |  |  |
| Maternal cortisol slope | 0.06 | 0.05 | .29 | 0.14 | 0.05 | **< .01** | 0.10 | 0.04 | **< .01** | 0.02 | 0.05 | .64 | -0.00 | 0.06 | .99 | 0.00 | 0.06 | .98 |
|  |  |  |  |  |  |  |  |  |  |  |  |  |  |  |  |  |  |  |
| Deviance | 2275.09 |  |  | 2237.47 |  |  | 4507.23 |  |  | 2294.45 |  |  | 2737.04 |  |  | 2840.40 |  |  |
| **Child physical neglect** |  |  |  |  |  |  |  |  |  |  |  |  |  |  |  |  |  |  |
| Intercept | 22.71 | 0.65 | **< .001** | 21.87 | 0.52 | **< .001** | 21.50 | 0.44 | **< .001** | 5.80 | 0.58 | **< .001** | 6.72 | 0.35 | **< .001** | 6.23 | 0.38 | **< .001** |
| Age child |  |  |  |  |  |  | 1.58 | 0.29 | **< .001** |  |  |  |  |  |  | 1.40 | 0.45 | **< .01** |
| Measurement time | -0.00 | 0.00 | **< .001** | -0.00 | 0.00 | **< .001** | -0.00 | 0.00 | **< .001** | 1.76 | 0.52 | **< .001** | 0.32 | 0.16 | **.046** | 0.04 | 0.01 | **< .001** |
| Measurement time squared |  |  |  |  |  |  |  |  |  | -0.36 | 0.10 | **< .001** | -0.08 | 0.02 | **< .001** | -0.00 | 0.00 | **< .001** |
| Maternal education | -0.19 | 0.19 | .31 | 0.30 | 0.15 | **.047** | 0.07 | 0.12 | .57 | -0.03 | 0.17 | .87 | 0.11 | 0.19 | .59 | 0.15 | 0.18 | .40 |
| Physical neglect | 0.06 | 0.13 | .63 | -0.01 | 0.10 | .95 | 0.02 | 0.08 | .81 | -0.05 | 0.12 | .71 | 0.07 | 0.14 | .62 | 0.00 | 0.12 | .99 |
| Psychopathology | 0.30 | 0.28 | .28 |  |  |  | 0.18 | 0.18 | .32 | -0.07 | 0.26 | .79 |  |  |  | -0.13 | 0.28 | .65 |
| Breastfeeding |  |  |  | 0.01 | 0.05 | .79 | -0.00 | 0.04 | .96 |  |  |  | 0.04 | 0.06 | .57 |  |  |  |
| Maternal cortisol slope | 0.06 | 0.05 | .30 | 0.14 | 0.05 | **< .01** | 0.10 | 0.04 | **< .01** | 0.03 | 0.05 | .62 | -0.00 | 0.06 | .97 | 0.00 | 0.06 | .95 |
|  |  |  |  |  |  |  |  |  |  |  |  |  |  |  |  |  |  |  |
| Deviance | 2274.68 |  |  | 2236.87 |  |  | 4506.90 |  |  | 2293.59 |  |  | 2736.48 |  |  | 2840.14 |  |  |
| **Child emotional neglect** |  |  |  |  |  |  |  |  |  |  |  |  |  |  |  |  |  |  |
| Intercept | 22.74 | 0.66 | < .001 | 21.85 | 0.52 | **< .001** | 21.50 | 0.44 | **< .001** | 5.80 | 0.58 | **< .001** | 6.72 | 0.36 | **< .001** | 6.22 | 0.38 | **< .001** |
| Age child |  |  |  |  |  |  | 1.58 | 0.29 | **< .001** |  |  |  |  |  |  | 1.40 | 0.45 | **< .01** |
| Measurement time | -0.00 | 0.00 | < .001 | -0.00 | 0.00 | **< .001** | -0.00 | 0.00 | **< .001** | 1.76 | 0.52 | **< .001** | 0.32 | 0.16 | **.046** | 0.04 | 0.01 | **< .001** |
| Measurement time squared |  |  |  |  |  |  |  |  |  | -0.36 | 0.10 | **< .001** | -0.08 | 0.02 | **< .001** | -0.00 | 0.00 | **< .001** |
| Maternal education | -0.18 | 0.19 | .30 | 0.15 | 0.15 | **.045** | 0.07 | 0.12 | .55 | -0.03 | 0.17 | .85 | 0.12 | 0.19 | .55 | 0.13 | 0.18 | .45 |
| Emotional neglect | 0.05 | 0.06 | .38 | -0.05 | 0.05 | .33 | -0.00 | 0.04 | .97 | -0.00 | 0.06 | .96 | -0.01 | 0.07 | .83 | -0.04 | 0.07 | .53 |
| Psychopathology | 0.30 | 0.27 | .27 |  |  |  | 0.18 | 0.18 | .32 | -0.06 | 0.26 | .79 |  |  |  | -0.13 | 0.27 | .64 |
| Breastfeeding |  |  |  | 0.01 | 0.05 | .82 | -0.00 | 0.04 | .92 |  |  |  | 0.03 | 0.06 | .63 |  |  |  |
| Maternal cortisol slope | 0.06 | 0.05 | .30 | 0.14 | 0.04 | **< .01** | 0.10 | 0.04 | **< .01** | 0.02 | 0.05 | .64 | -0.00 | 0.06 | .99 | 0.00 | 0.06 | .97 |
|  |  |  |  |  |  |  |  |  |  |  |  |  |  |  |  |  |  |  |
| Deviance | 2275.66 |  |  | 2237.44 |  |  | 4508.48 |  |  | 2295.28 |  |  | 2738.16 |  |  | 2840.92 |  |  |
